# Repetitive Treatment with Volatile Anesthetics Does Not Affect the In Vivo Plasma Concentration and Composition of Extracellular Vesicles in Rats

**DOI:** 10.3390/cimb43030137

**Published:** 2021-11-13

**Authors:** Christian Bleilevens, Christian Beckers, Alexander Theissen, Tamara Fechter, Eva Miriam Buhl, Johannes Greven, Sandra Kraemer, Sebastian Wendt

**Affiliations:** 1 Department of Anesthesiology, Medical Faculty, University Hospital RWTH Aachen, 52074 Aachen, Germany; atheissen@ukaachen.de (A.T.); tfechter@ukaachen.de (T.F.); 2 Department of Intensive Care Medicine, Medical Faculty, University Hospital RWTH Aachen, 52074 Aachen, Germany; cbeckers@ukaachen.de (C.B.); skraemer@ukaachen.de (S.K.); 3 Institute of Pathology, Electron Microcopy Facility, University Hospital RWTH Aachen, 52074 Aachen, Germany; ebuhl@ukaachen.de; 4 Department of Orthopaedics, Trauma and Reconstructive Surgery, University Hospital RWTH Aachen, 52074 Aachen, Germany; jgreven@ukaachen.de; 5 3CARE-Cardiovascular Critical Care & Anesthesia Evaluation and Research, 52074 Aachen, Germany

**Keywords:** anesthetic-induced preconditioning (AIP), extracellular vesicle (EV), in vivo, nanoparticle tracking analysis (NTA), flotillin, CD63

## Abstract

Background: Anesthetic-induced preconditioning (AIP) with volatile anesthetics is a well-known experimental technique to protect tissues from ischemic injury or oxidative stress. Additionally, plasmatic extracellular vesicle (EV) populations and their cargo are known to be affected by AIP in vitro, and to provide organ protective properties via their cargo. We investigated whether AIP would affect the generation of EVs in an in vivo rat model. Methods: Twenty male Sprague Dawley rats received a repetitive treatment with either isoflurane or with sevoflurane for a duration of 4 or 8 weeks. EVs from blood plasma were characterized by nanoparticle tracking analysis, transmission electron microscopy (TEM) and Western blot. A scratch assay (H9C2 cardiomyoblast cell line) was performed to investigate the protective capabilities of the isolated EVs. Results: TEM images as well as Western blot analysis indicated that EVs were successfully isolated. The AIP changed the flotillin and CD63 expression on the EV surface, but not the EV concentration. The scratch assay did not show increased cell migration and/or proliferation after EV treatment. Conclusion: AIP in rats changed the cargo of EVs but had no effect on EV concentration or cell migration/proliferation. Future studies are needed to investigate the cargo on a miRNA level and to investigate the properties of these EVs in additional functional experiments.

## 1. Introduction

Preconditioning is a commonly used experimental method to protect organs and tissues from injury, related to hypoxic or ischemic insults [1]. Anesthetic-induced preconditioning (AIP) is one of the most frequently used strategies, besides hypoxic or ischemic preconditioning (IPC) [2]. Cardiovascular and cerebrovascular diseases, such as myocardial infarction and stroke, are the leading causes of death in the world related to prolonged hypoxic or ischemic organ injury (according to the WHO). A variety of experimental in vitro and in vivo models target the heart and the brain as the organs of interest in different animal- or cell-culture AIP or IPC models. In detail, in vitro AIP or IPC models are characterized as repetitive incubation cycles of cardiomyocyte cell cultures; for instance, with volatile anesthetics [3] or noble gases [4]. Additionally, hippocampal slice cultures preconditioned prior to traumatic brain injury represent common experimental settings [5]. For in vivo models, clinically relevant scenarios, such as cardiopulmonary resuscitation (CPR) [6,7], stroke [8] or myocardial infarction [9] in different species represent the basis to investigate the preconditioning hypothesis.

This method’s translation into clinics is debated. Depending on the scenario, the choice of volatile anesthetic agents in cardiovascular surgeries, such as coronary artery bypass graft surgery [10] or general cardiac surgery [11], provides benefits in terms of cardio protection, as biomarkers such as troponin have shown themselves to be reduced in comparison to total intravenous anesthesia. In 2017, the European Association of Cardiac Surgery guidelines declared the use of volatile anesthetics superior during coronary artery bypass surgery compared to intravenous anesthesia [12]; however, in 2019, no benefits concerning the clinical outcome after cardiac surgery were declared in a large meta-analysis including 5400 patients [13]. For neurological damage scenarios, such as stroke, the benefit of AIP and general preconditioning methods is much clearer; preconditioning increases brain resistance against acute brain injury via modulation of neuroinflammation [14]. However, for a real translation of an experimental AIP scenario into clinics, evidence-based data about benefits or disadvantages for different scenarios are still missing. The exact mechanisms, how the described preconditioning methods provide their effects, are not yet fully understood. Mechanisms such as the elevated expression of several mRNAs or the activation of protective kinases are described in the literature [4,15]. In recent years, research in the field of extracellular vesicles (EVs) has experienced rapidly growing interest, especially as possible mediators of protective molecules [3,16]. EVs are small particles secreted by nearly every cell type of a mammalian organism, carrying different cargos, such as DNA [17,18], RNA/miRNA [19,20] and proteins [21]. EVs are generally distinguished by their size and generation [22]. The smallest ones are exosomes derived from inward budding of the cellular membrane and released by the fusion of multi-vesicular bodies with the plasma membrane [23]. Microvesicles, the second biggest population of EVs, are generated by budding from the plasma membrane, whereas the biggest type of vesicles, apoptotic bodies, are formed in the post-apoptosis phase [24]. EVs have been described as initiators of tissue restoration processes after cardiovascular and cerebrovascular insults, and are upregulated after anesthetic preconditioning of cardiomyocyte cultures [3,25,26]. In general, EVs mediate their protective properties through different mediators such as miRNAs or proteins [16]. Furthermore, EVs in different concentrations were detected after colorectal cancer resections in patients treated with sevoflurane or propofol [27]. Additionally, the mediation of cardioprotective effects by remote ischemic preconditioning was also described as being related to EVs [28].

In cerebrovascular events, EVs, derived from mesenchymal stem cells (MSCs), have been shown to initiate restorative effects after stroke. EVs seem to play a crucial role in functional-, grey- and white-matter repair via initiation of neuro- and angiogenesis in the penumbra zone of the ischemic brain [26,29,30]. IPC has been described to trigger the release of beneficial and protective EVs in the scenario of ischemic brain injury [31].

However, it remains unclear whether in vivo AIP might mediate the expression of presumably protective EVs against cardiovascular und cerebrovascular injuries.

Therefore, we investigated the expression of EVs after repetitive inhalation of two commonly used volatile anesthetics (sevoflurane, isoflurane) in healthy rats, and examined whether four or eight weeks of AIP might change the composition and concentration of circulating EVs in comparison to baseline measurements.

## 2. Materials and Methods

### 2.1. Animals

All experiments were performed in accordance with the German Federal Law regarding the protection of animals and the DIRECTIVE 2010/63/EU on the protection of animals used for scientific purposes. Official permission was obtained from the governmental care and use committee (LANUV, Recklinghausen, NRW, Germany) (file reference: 81-02.04.2017.A387).

A total of 20 male Sprague Dawley rats (Charles River, Cologne) weighing 348 ± 17 g at the beginning of the experiments were used. The animals were kept in the Institute for Laboratory Animal Science (University Hospital Aachen Germany; certified according to DIN ISO 9001/2015 QM) and group-housed in Type 2000 rat filter top cages (Tecniplast, Hohenpeißenberg, Germany) under specific pathogen-free conditions according to the guidelines of the Federation of European Laboratory Animal Science Associations [32]. They were allowed to acclimate to the new surroundings for 7 days, before the experiments started.

### 2.2. Experimental Groups

The animals were assigned to three different main groups. One main group received repetitive inhalation of isoflurane in an appropriate Plexiglas inhalation chamber (Leica Biosystems, Wetzlar, Germany), divided into a four-week subgroup (ISO4, n = 4), and an eight-week subgroup (ISO8, n = 4). The second main group received repetitive inhalation of sevoflurane, also divided into a four-week subgroup (SEVO4, n = 4) and an eight-week subgroup (SEVO8, n = 4). In the control group (CON), four animals received no inhalation, but they were placed for the same time of the inhalation procedure into an empty cage, to simulate the handling stress during transfer into the inhalation chamber. From all animals, one single blood sample was withdrawn before the inhalation protocol started, which was used as pooled baseline (BL) sample. The inhalation protocol was equal for all groups, and consisted of three times a week (Monday, Wednesday, Friday) of 30 min isoflurane- or sevoflurane inhalation, or sitting in an empty cage without inhalation. The time was started as soon the animals fell asleep in a 4 Vol% isoflurane or sevoflurane atmosphere, mixed with room air, for anesthesia induction. For maintenance of anesthesia, concentrations were reduced to 1.3 Vol% isoflurane, and 2.3 Vol% sevoflurane, respectively, according to the optimal minimum alveolar concentration (MAC) values for volatile anesthetics in adult rats, reported by Orliaguet et al. in 2001 [33]. After the inhalation procedure, the rats were transferred back to their cages and the housing rooms within the facility.

### 2.3. Catheterization for Baseline Measurement, and Final Blood Draw

Prior to the start of the inhalation phase of 4, or 8 weeks, each animal received 45 min anesthesia via subcutaneous (s.c.) injection of 150 μg Meditomedin (Vetoquinol, Ismaningen, Germany), 2 mg Midazolam (ratiopharm, Ulm, Germany) and 50 μg Fentanyl (Rotexmedica, Trittau, Germany) per kg bodyweight, to cannulate the left sided femoral vein, using appropriate intravascular tubing (#SX03, ID 0.4 mm, A. Hartenstein Inc., Würzburg, Germany). Via the venous catheter, 1 mL of whole blood was drawn into 0.5 mL citrated monovettes (Sarstedt, Nümbrecht, Germany). Afterwards, the catheter was removed, the vein was ligated permanently, wounds were closed in a double layer using surgical thread (4-0, Johnson & Johnson, Neuss, Germany) and the animals received an injection of 0.2% Ropivacain (Naropin, Astra Zeneca Inc., Wedel, Germany), as local anesthesia to prevent pain until full recovery from anesthesia. After four or eight weeks of anesthesia inhalation, animals were sacrificed by i.p. bolus injection of 800 mg/kg BW Pentobarbital Sodium (Narcoren, Merial inc, Hallbergmoos, Germany), and heart was punctured for the final blood withdrawal of 3–4 mL of blood into citrated monovettes. Organs (Heart, Lungs, Liver, Kidneys) were extracted, as soon as no heartbeat was detectable.

### 2.4. Isolation of EVs

The citrated whole blood samples were initially centrifuged at room temperature (RT) for 10 min at 2000× *g*, and the supernatant was transferred into a new reaction tube for further centrifugation at RT for 30 min at 2000× *g*. The supernatant was stored at −80 °C until all animals finished the inhalation protocol and all blood samples were collected. The isolation of EVs was performed according to a centrifugation protocol described by Qiao et al. [32]. Briefly, the frozen blood plasma samples were thawed at RT, filled until 1 mL total volume with sterile filtrated phosphate-buffered saline (PBS, Thermofisher Scientific, Langerwehe, Germany), centrifuged at 4 °C for 90 min at 20,000× *g*. The supernatant was discarded, the pellet was resuspended in 1 mL of sterile PBS and centrifuged again (4 °C, 90 min, 20,000× *g*). The resulting pellet was resuspended in 200 µL sterile filtrated PBS and used for the subsequent analysis.

### 2.5. Western Blot

Antibodies for the detection of specific proteins which are associated with EVs were used. All antibodies were purchased from BD Biosciences (ALIX: BD611620, flotillin-1: BD610821, CD63: BD551458, BD Biosciences Inc., Heidelberg, Germany). The secondary anti-mouse antibody was purchased from GE healthcare (#Na931V, GE Healthcare, Munich, Germany).

For each sample, 38 µL was used for a standard gel electrophoresis, using 10% Acrylamide Stainfree-gels (# 1610172, BioRad, Feldkirchen, Germany) and 5 µL of a positive control, produced by cardiomyocyte stem cell culture lysate (H9C2 cells, CRL-1446, ATCC, Manassas, VA, USA), as described previously [3]. The samples for the detection of ALIX and Flotellin-1 were prepared as follows: 52 µL sample, 8 µL DTT (R0861, ThermoFisher Scientific, Langerwehe, Germany), 20 µL LDS (B0008, ThermoFisher Scientific, Langerwehe, Germany). For the detection of CD63, the same protocol was used, except for the usage of sterile filtrated aqua dest instead of DTT. The normalization of the single EV markers was performed using the total protein amount of the corresponding sample, visualized within the Acrylamide Gel after the electrophoresis and prior to the blotting procedure by Stainfree technology, as described previously [3].

The separated proteins were transferred to a PVDF membrane using a semi-dry-turbo-blotting system (#1704150, BioRad, Feldkirchen, Germany). After the blotting procedure, unspecific binding sites were blocked, using a mixture of 2.5% milkpowder (T145.3, CarlRoth, Karlsruhe, Germany) and 2.5% bovine serum albumin (BSA, 8076.1, CarlRoth, Karlsruhe, Germany) in TBS-T buffer (1061.1, CarlRoth, Karlsruhe, Germany). Afterwards, the membranes were incubated with the primary antibodies (ALIX: 1:500 in 1% milkpowder, flotilin-1: 1:500 in 1% milkpowder, CD63: 1:500 in 1% BSA) according to the manufacturer’s specifications, followed by repetitive washing steps and incubation with secondary antibody (1:5000 in 1% milkpowder), labeled with horseradish peroxidase (HRP). Chemiluminescent signals were detected after incubation of the membrane with ECL-Reagent (A38554, ThermoFisher, Langerwehe, Germany) using the ChemiDoc Imaging system (BioRad, Karlsruhe, Germany). Densitometrical analysis of the protein bands intensity was performed using image lab software (BioRad) and displayed as integrated density values (IDV).

### 2.6. Nanoparticle Tracking Analysis (NTA)

Size and concentration of isolated EVs were quantified by NTA using the NanoSight NS300 (Malvern Panalytical Ltd., Malvern, UK) equipped with a sCMOS camera and a Blue488 laser module. All samples were diluted 1:50–1:100 in DPBS (D8662, Sigma Aldrich, Taufkirchen, Germany) to a final volume of 1 mL. Capture settings were used according to the manufacturer’s manual, camera focus was adjusted by autofocus and camera level was set to 14. For each measurement, 3 60 s videos were captured. The videos were analyzed by the NanoSight Software NTA 3.2 Dev Build 3.2.16 with a detection threshold of 7.

### 2.7. Transmission Electron Microscopy (TEM)

Native EVs were incubated for 5 min on glow-discharged formvar carbon-coated grids (Nickel Grid 200 mesh, Electron Microscopy Sciences, Hatfield, PA, USA). Grids were washed three times with H_2_O. Negative staining was performed with 0.5% uranyl. Excess liquid was removed using filter paper. The grids were air dried for 10 min. Samples were imaged using a LEO 906 E transmission electron microscope (Zeiss, Oberkochen, Germany), operated at an acceleration voltage of 60 kV.

### 2.8. Cell Migration Assay (Scratch Assay)

As previously described, a cell migration assay on cardiomyoblast cell line (H9C2) was performed [3]. Briefly, a confluent monolayer of H9C2 cells (CRL-1446, ATCC, Manassas, VA, USA) was scratched with a 200 µL pipette tip, then the cells were washed in PBS and incubated with 5 × 10^8^ EVs/mL in 1% EV-depleted (fetal bovine serum) FBS medium for 24 h, and PBS was used as negative control. The cell cultures were fixed in 4% formaldehyde and stained with Fluoromount g (DAPI, SouthernBiotech Birmingham, Al, USA). An EVOS cell imaging system (Thermo Fisher Scientific, Waltham, MA, USA) was used to take digital images of the stained cell cultures. ImageJ Freeware (imagej.nih.gov/version 1.8.0, National Institutes of Health, Bethesda, MD, USA) was used to quantify differences in the scratch areas between the experimental groups.

### 2.9. Statistical Analysis

Statistical analysis was performed using GraphPad Prism 8 software package (GraphPad Software Inc, La Jolla, CA, USA). A one-way analysis of variance (ANOVA) was applied, followed by Tukey post-test correction for multiple comparison tests, after performing a Shapiro–Wilk normality test to compare the IDV values of the Western blot analysis for the concentration and size measurements via NTA between the groups. Data are presented as mean ± SD and a *p* value < 0.05 was considered statistically significant.

## 3. Results

Volatile anesthetics have an effect on the composition of EVs derived from numerous cell types such as cardiomyocytes and fibroblasts [3]. Nevertheless, these experiments were performed in vitro, and it still remains unclear whether these stimuli lead to a similar effect in vivo. We want to highlight that the specific source of the investigated EVs is not clear since we analyzed blood-derived EVs, resulting in a mixture of EVs from different cell types.

### 3.1. TEM

**Figure 1:**
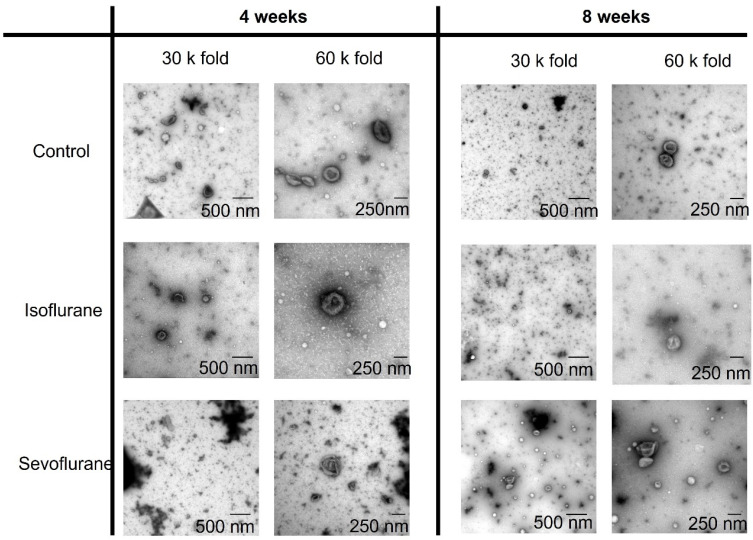
Extracellular vesicles (EVs) isolated from plasma samples after 4 and 8 weeks of preconditioning in the isoflurane, sevoflurane or control group.

Electron microscopy of the 4- and 8-week plasma samples could prove the clean isolation of EVs from plasma samples. No differences in the concentration or the appearance of EVs between the experimental groups were shown (Figure 1). However, since this method is not well suited for characterizing the size and concertation of EVs on a larger scale, these characteristics were investigated by NTA.

### 3.2. Western Blot

**Figure 2:**
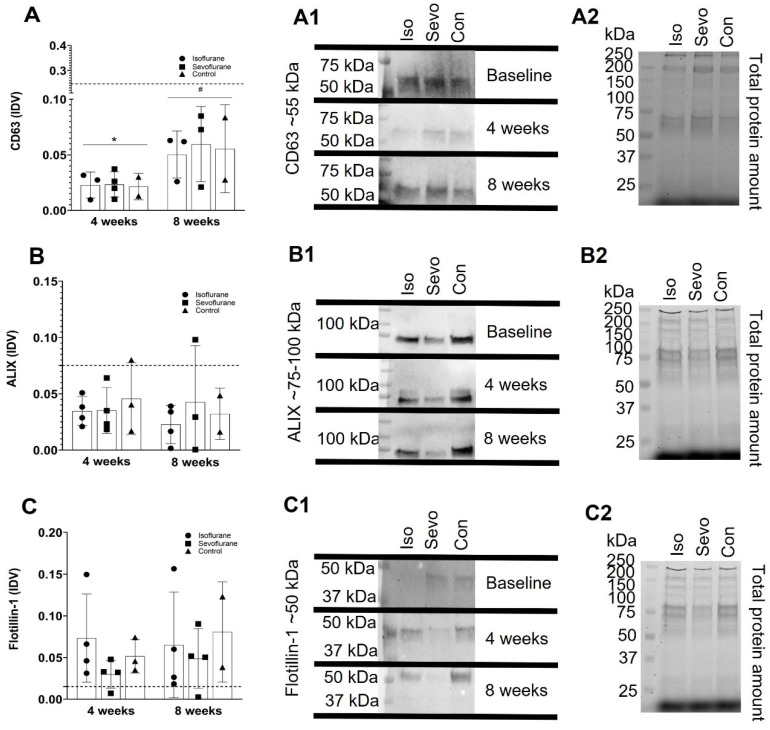
Integrated density values (IDV) of CD63 (**A**), ALIX (**B**), and flotillin-1 (**C**), with the corresponding and representative protein bands of CD63 (**A1**). ALIX (**B1**), and flotillin-1 (**C1**) from the Western blot analysis, normalized to the corresponding total protein amount visualized in the acrylamide gels (**A2**,**B2**,**C2**) comparison between the groups and vs. baseline values (dotted line), after 4 and 8 weeks of AIP. * *p* < 0.05 vs. BL/# *p* < 0.05 vs. BL.

To further characterize the isolated EVs and to investigate whether the treatment had any effect on the expression of EV specific markers, Western blot analysis was performed. The amount of CD63-positive EVs was decreased significantly at 4 and 8 weeks in comparison to BL measurements. No differences could be detected between the groups (Figure 2A). In contrast, the amount of ALIX-positive EVs remained stable over 8 weeks, without differences in comparison to BL; no differences between any of the groups were detected (Figure 2B). The amount of Flotellin-1 positive EVs increased after 4 weeks in comparison to BL and remained stable until the end of the experiments after 8 weeks, without any differences between the experimental groups (Figure 2C).

### 3.3. NTA

**Figure 3:**
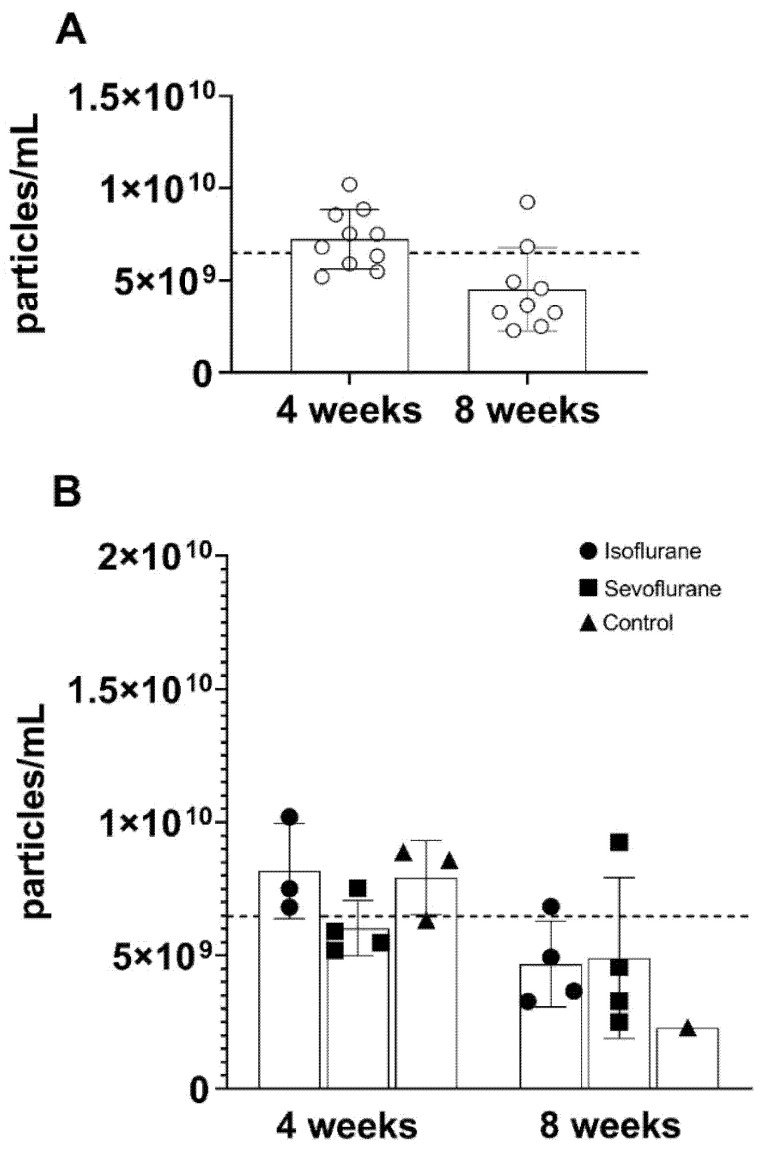
Particle concentration (particles/mL), did not differ significantly over time (**A**). According to the experimental group, the particle concentration was not different between the groups (**B**). Baseline values were indicated as dotted line.

To investigate the concentration and size of the isolated EVs in all groups, we performed nanoparticle tracking analysis. The particle concentration showed no significant differences between the treatment after 8 weeks and the BL measurements, independent of the experimental group (Figure 3A). Additionally, between the experimental groups, no significant changes in particle concentration were detected (Figure 3B).

**Figure 4:**
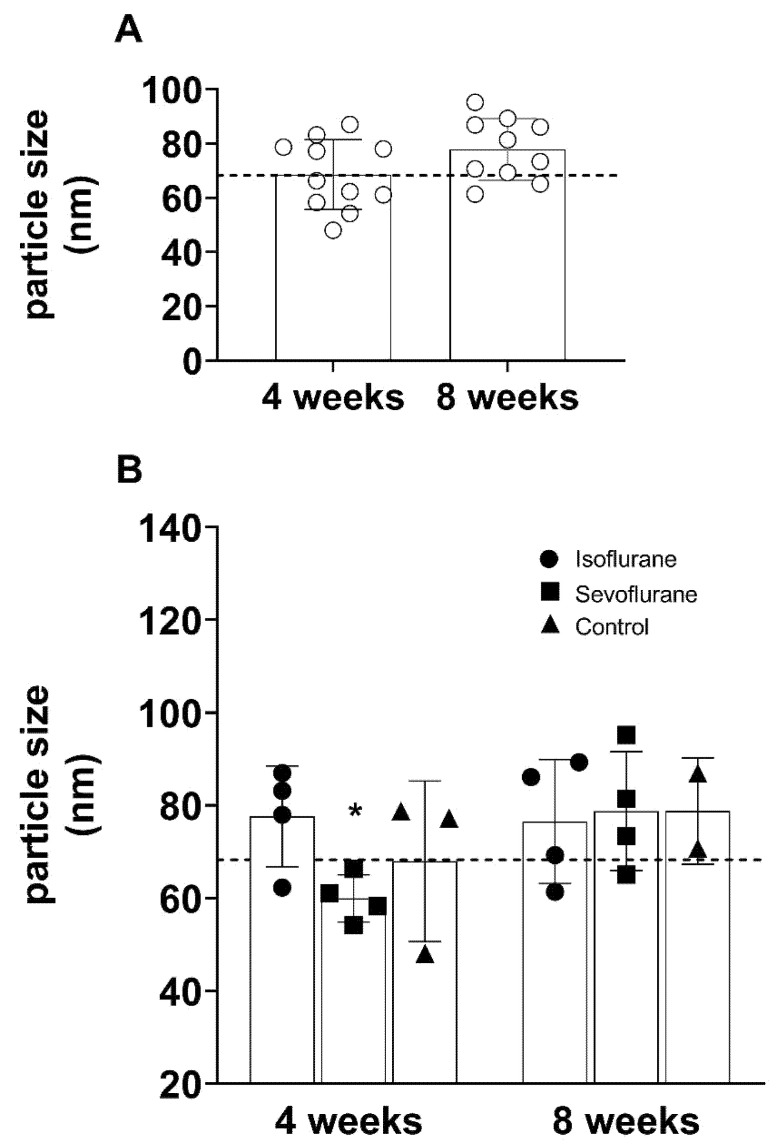
Particle size (nm), did not differ significantly over time (**A**). According to the experimental group, the particle size was reduced in the sevoflurane group after 4 weeks in comparison to isoflurane, and the measurement after 8 weeks (**B**). Baseline values are indicated as dotted line. * *p* < 0.05 vs. isoflurane 4 weeks and sevoflurane 8 weeks.

The particle size of the detected EVs did not change over time, if measured independent from the group (Figure 4A). However, if analyzed according to the experimental group, the size of the EVs in the sevoflurane group was significantly reduced in comparison to the isoflurane group and the 8 week results, but not in comparison to the BL values (Figure 4B).

### 3.4. Scratch Assay

**Figure 5:**
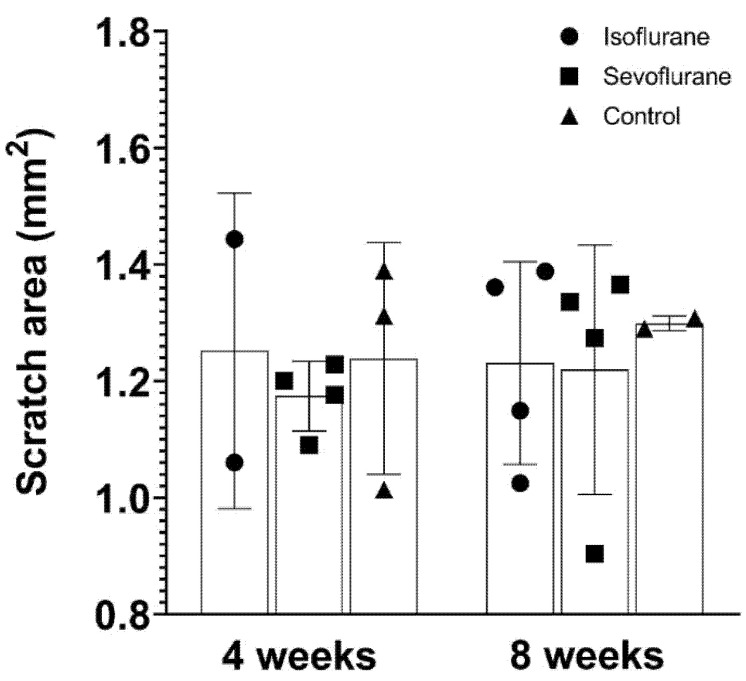
The scratch assay was performed on H9C2 cell cultures using a standardized cell scraper, resulting in a distinct scratch area (mm^2^), which was not significantly enlarged or reduced by any of the extracellular vesicle (EV) populations in the experimental groups.

The potential benefits of the different EV populations concerning improvements in cell proliferation and migration after performing a cell scratch did not show any differences between the experimental groups. The size of the scratch area was not influenced when cells were incubated with EVs previously purified from the treated animals (Figure 5).

## 4. Discussion

AIP is one of the most frequently used strategies in experimental preconditioning, but the interplay between AIP and the resulting EVs population, which has been described as potential transmitter of beneficial AIP effects, is not completely understood. Therefore, the aim of this study was to investigate the potential effect of repetitive ISO or SEVO anesthesia in rats on the EV population in comparison to non-treated animals. Electron microscopy images indicated that we were able to isolate EVs from rat plasma in all groups. Furthermore, we could show that repetitive administration of volatile anesthetics in general over 4 or 8 weeks led to significantly reduced CD63-, and significantly increased flotillin-positive EVs in comparison to baseline levels, without any differences between the experimental groups. The only relevant effect of AIP with SEVO could be shown on EV size, which was significantly smaller in this group. For ISO or the control group, no significant effects in any of the analytical methods could be shown. Additionally, the EV concentration did not change between any of the groups. Finally, we tested the isolated EVs from in vivo AIP for functional effects in an in vitro cell migration assay. No significant effect was shown in any of the groups.

In contrast to in vitro studies, observing the AIP effect for a distinct cell line, including EV isolation from supernatant, the in vivo AIP applied in this study represents the systemic EV population isolated from plasma. As shown previously, the systemic EV population from rat serum or plasma after myocardial infarction [34], from type two diabetes rats [35], or after stroke [36], provides a broad range of beneficial and organ protective effects. The EVs from myocardial infarction rats, for instance, activate the protein kinase B (AKT), which promotes pro-survival and anti-apoptotic signaling in any tissue or cell. Type two diabetes-derived EVs provide neuroprotective properties, and stroke derived EVs protect neurons against ischemia reperfusion injury.

However, the translation of a preconditioning scenario based on the collection of EVs from myocardial infarction or stroke patients, and later application as protective agents, is not realistic to implement. Even the prolonged repetitive AIP scenario, as presented in this study, is not realistic. Therefore, the available data about preconditioning in clinical scenarios are limited to short 3 min sequences of AIP before and during cardiac surgery, for example [37], or to a replacement of propofol by desflurane at 30 min before the onset of ischemia during hepatectomy, for example, in [38]. Nevertheless, both settings show beneficial effects in reducing the inflammatory response.

Thus, the idea of this study was to isolate EVs from a non-invasive AIP scenario for repetitive treatment with volatile anesthetics in rats, in contrast to invasive experimental models (myocardial infarction or stroke), to achieve a potential organ-protective EV population, or at least one that acts systemically, as the available literature concerning AIP let us assume.

To justify in vivo experiments of the EVs derived from this study in ischemia reperfusion models, such as myocardial infarction or stroke, a preliminary step would be to prove that the different stimuli result in treatment-dependent differences regarding the vesicle population. This could be differences in size and concentration, as previously used to distinguish between EVs from M0 or M1 macrophages, for example [39], and/or differences in the EV cargo, such as proteins or miRNAs; for instance, CD63, CD81 receptors, flotillin or HSP70 expression on the EV surface, all of which provide evidence regarding different EV populations [3].

We could show a significant reduction in CD63-positive EVs after 4 and 8 weeks of ISO or SEVO treatment in comparison to the BL level, but the differences in the control group were not significant; thus, the effect could not be related to AIP. The reduction in CD63 EVs in all the experimental groups was only accompanied in the SEVO group by a significant reduction in EV size, at least after 4 weeks of treatment.

Although CD63 generally used as a surface marker to identify EVs [40], there are also reports about different miRNA cargos in CD63-positive EVs in comparison to CD47-positive EVs, such as those with potentially different functions [41].

Whether the observed change in the CD63 concentration in EVs or the size reduction contribute to any functional properties of the EVs generated in our setting should be evaluated by a cell migration assay on H9c2 cells; this approach was suitable in our previous study to prove increased cell proliferation effects related to increased EV counts from cell cultures treated with volatile anesthetics or hypoxia [3]. However, we were not able to observe any cell migration or proliferation in the scratch assay for any of the experimental groups. Since numerous studies have shown that EVs are generally able to induce cell migration or proliferation, it remains unclear why we were not able to observe any effect on the cellular level [16,42,43]. Other viability assays such as a tube formation assays or general stress/apoptosis assays might also have been suitable to investigate potential protective properties of EVs isolated from treated animals, but the EV concentration was too low to perform more tests.

Nevertheless, we were able to detect altered cargo in EVs from AIP-treated animals but were not able to prove a beneficial or detrimental effect. Both ways of action are possible, as not only the previously described beneficial effects might be mediated by EVs Cargo, but proinflammatory processes could also be mediated by proinflammatory cytokines, or mediators such as C-reactive protein as EV cargo, and thus play a major role during systemic inflammatory processes such as sepsis [44].

The study has, in our understanding, several limitations. The available blood volume from rats was not sufficient to include a further analysis, such as miRNA isolation from the vesicles, as described in our previous study [3], or additional cell viability assays, which could not be performed due to too low of an EV yield. Additionally, we had a small “n” in each of the groups; however, it was unlikely that a larger n would have turned the results towards more relevant insights, especially concerning functional testing.

## 5. Conclusions

In conclusion, we show that repetitive treatment of rats over 4 and 8 weeks with the volatile anesthetics isoflurane or sevoflurane does not change the plasma EV population significantly. The beneficial effects for cell proliferation and/or migration of EVs derived from in vitro AIP could not be transferred to EVs from our in vivo AIP model. Further studies are needed to investigate differences in the EV composition from the different groups on the miRNA level. Different assays on the cellular level will provide further insight as to whether the isolated EVs from in vivo AIP models mediate beneficial properties to different cells, such as EVs from in vitro AIP.

## Data Availability

The data presented in this study are available on request from the corresponding author.
